# Breast cancer recurrence: factors impacting occurrence and survival

**DOI:** 10.1007/s11845-022-02926-x

**Published:** 2022-01-25

**Authors:** Donald Courtney, Matthew G. Davey, Brian M. Moloney, Michael K. Barry, Karl Sweeney, Ray P. McLaughlin, Carmel M. Malone, Aoife J. Lowery, Michael J. Kerin

**Affiliations:** Department of Surgery, National University of Ireland, Galway, H91YR71 Republic of Ireland

**Keywords:** Breast cancer, Metastasis, Personalized medicine, Recurrence

## Abstract

**Background:**

Breast cancer mortality has decreased due to improved screening and treatment options. Nevertheless, 25–30% of patients develop disease recurrence and die from the disease dissemination. Patients who develop metastatic disease represent a heterogeneous group and management plans are dependent on molecular subtype, disease burden and metastatic site.

**Aim:**

To determine predictive clinicopathological factors of disease recurrence and their impact on survival in the molecular era.

**Methods:**

Consecutive patients who breast cancer developed recurrence at our tertiary referral centre between 2000 and 2015 were included. Clinicopathological and treatment data were assessed using descriptive statistics. Oncological outcome was assessed using Cox regression and Kaplan Meier analyses.

**Results:**

Two hundred sixty-five consecutive patients who developed breast cancer recurrence were included; median age at metastasis was 59.3 years (range 27–87 years), and median time to recurrence (TTR) was 47.7 ± 38.5 months (range 3.0–194.3 months). Survival was 24.2% (64/265) 53.2% were luminal A (LABC) (141/265), 18.5% were luminal B (LBBC) (49/265), 18.5% were triple negative (TNBC) (49/265), and 9.8% were human epidermal growth factor receptor-2 overexpressing (HER2 +) (26/265). TTR for patients with LABC was 56.0 ± 41.3 months, LBBC was 48.4 ± 41.1 months, TNBC was 26.9 ± 28.5 months and HER2 + was 34.3 ± 21.8 months. Increased grade (*P* < 0.001), Nottingham Prognostic Indices (*P* < 0.001), TNBC (*P* < 0.001), HER2 + subtype (*P* < 0.001) and receiving targeted therapy *(P* = 0.006) predicted shorted TTR. Estrogen receptor positivity (*P* < 0.001), progesterone receptor positivity (*P* = 0.010), invasive lobular carcinoma (*P* = 0.009) and receiving endocrine therapy (*P* = 0.001) predicted longer TTR.

**Conclusion:**

Readily available clinicopathological factors predict risk of metastatic dissemination. Developing a tailored program to identify patients at risk of recurrence is crucial in controlling metastatic dissemination of breast cancer.

## Introduction

Breast cancer is a heterogenous disease, with varying molecular properties, multimodal therapeutic strategies and expected prognoses [1]. At present, breast cancer is one of the most frequently diagnosed malignancies worldwide, accounting for 23% of female cancer diagnoses, as well as a leading cause of cancer-related mortality in women, responsible for 14% of cancer deaths [2]. In the setting of recurrence, breast cancer is often considered incurable, and in spite of enhanced therapeutic strategies and surveillance, 5–10% of patients will have metastatic disease at initial presentation, while a further 20% develop disease recurrence a later stage [2]. Traditionally, the anticipated survival time for patients who succumbed to metastatic disease was variable, ranging from 9 months to 3 years [3], and carried poor prognoses [4].

Historically, only a minority of patients with metastatic breast cancer survived beyond 10 years [5]. In recent times, expected survival has greatly improved, with data from the Surveillance, Epidemiology and End Results (SEER) suggest 5-year survival in the setting of metastatic disease to be 27% [6]. The reasons for the improved outcome include an increased appreciation of the biological properties driving tumour growth and dissemination, enhanced diagnostics and novel adjuvant and endocrine treatment strategies [7, 8]. Nevertheless, we must acknowledge that recurrent breast cancer is a separate entity, presenting several challenges different to those presenting with local disease. Moreover, breast cancer recurrence presents a plethora of idiosyncratic features contributing to outcome and is managed according to specific protocols, which differ greatly from those used in the primary setting.

In the disease recurrence, the outcome is dependent on several clinicopathological and immunohistochemical (IHC) characteristics including increased tumour burden, shorter metastatic interval as well as general patient performance factors [9]. Moreover, oestrogen receptor positive (ER +) cancers have the tendency to metastasise to bone and are associated with favourable outcomes [5], likely due to the presence of steroid hormone receptors providing targets for endocrine agents, from which there is typically the best treatment for establishing disease control, even in the setting of stage IV disease [10–12]. Recent manuscripts emphasise individual factors impacting outcome [13], and there is now an enhanced appreciation for prognostic clinicopathological parameters influencing recurrence and mortality. Moreover, these features are crucial in determining appropriate patient surveillance to address the presence of metastatic tumour recurrence and treatment/possible prevention strategies. Accordingly, the aim of the current study is to evaluate the patterns of disease recurrence and to assess the impact of clinicopathological and treatment characteristics on outcome in the those who develop breast cancer recurrence.

## Methods

### Study design and patient selection

This study was granted institutional review board approval from the Galway University Hospitals (GUH) Clinical Research Ethics Committee. A single centre, retrospective observational cohort study was undertaken. Data was obtained from a prospectively maintained institutional database that included patients who were treated for breast cancer between January 2000 and January 2015 at GUH, a tertiary referral centre serving the west of Ireland. Overall, 502 patients were recorded as having metastatic disease during the study period, of which 243 were diagnosed as having metastasis at initial presentation (M1). All of these patients who presented with M1 disease at diagnosis were excluded, leaving only those who were treated originally with curative intent and then suffered a disease recurrence. For the purpose of this study, only patients with complete clinicopathological details including treatment received both at primary diagnosis and subsequent disease recurrence were included. Detailed information regarding patient demographics, clinicopathological data, surgical management, adjuvant treatment regimens, disease recurrence and survival were collected using this database, and all data was cross-referenced with patient electronic and medical records.

### Data collection

The primary outcome of interest was overall survival (OS) which was defined as ‘the interval between diagnosis and death from any cause’. Invasive disease-free interval (DFI or iDFI) was defined as ‘the interval between diagnosis and distant recurrence of any type’. Clinicopathological characteristics were evaluated: demographic data (mean age at diagnosis, menopausal status at diagnosis), histopathological characteristics (histological subtype, Nottingham tumour grade, tumour size and staging, degree of nodal involvement and staging, Nottingham Prognostic Index (NPI), immunohistochemical tumour characteristics (e.g.; estrogen (ER) and progesterone receptor (PgR) status, human epidermal growth factors receptor-2 (HER2) status, etc.), site of metastasis and therapies received (neo)adjuvant chemotherapy, adjuvant radiotherapy, endocrine therapy, and targeted monoclonal antibodies (MAB).

### Histopathologic and immunohistochemical evaluation

ER and PgR status were determined using the Allred scoring system [14]. IHC was used to assess HER2 status; those scoring 2 + were submitted for fluorescence in situ hybridization (FISH) to confirm HER2 receptor status. The Elston-Ellis modification of Scraff-Bloom-Richardson grading system was applied to grade tumour specimens in accordance to the Nottingham Histologic Score system [15]. D2-40 staining was used to evaluate tumour lymphatic invasion, CD34 was used for vascular invasion and S-100 , ,and a broad spectrum keratin stain (AE1/AE3) was used for perineural invasion [16]. Ki-69 was evaluated using MIB1 antibody testing [17, 18]. Nottingham prognostic indices were graded in accordance with the original scoring system as described by Galea et al. [19]. Molecular subtypes were defined in accordance with Goldhirsch et al. at the 2013 St. Gallen international expert consensus [20].

### Statistical analysis

Statistical analysis was carried out using Statistical Package for Social Sciences™ (SPSS™) version 26. Descriptive statistics were used to assess the impact of clinicopathological details on recurrence in the form of metastatic disease with independent Student’s *t* test, Chi-squared, one-way ANOVA and Kruskal–Wallis tests as appropriate. Follow-up was recorded through a prospectively maintained database. The median and mean lengths of follow-up were calculated using the reverse Kaplan–Meier method [21]. Kaplan–Meier curves, the Log-rank (Mantel-Cox) test and Cox-regression were used to associate survival with clinical, pathological and IHC characteristics expressed as hazard ratios (HR) with 95% CIs. All tests of significance were 2-tailed, with *P* < 0.050 indicating statistical significance. Data was analysed using Statistical Package for Social Sciences™ (SPSS™) version 26.

## Results

### Clinicopathological characteristics

Two hundred and sixty-five consecutive patients were included in this study. The median age at diagnosis of disease recurrence was 59.3 years (range 27–87 years). Overall, 53.2% of patients had the Luminal A molecular subtype (LABC) (141/265), 18.5% had Luminal B (LBBC) (49/265), 18.5% had triple negative (TNBC) (49/265) and 9.8% had HER2 overexpressing (HER2 +) breast cancers (26/265). Clinicopathological and metastatic data are illustrated in Table 1.

**Table 1:** Clinicopathological data of patients who developed metastatic disease (*n* = 265)

	Luminal A (*n* = 141)	Luminal B (*n* = 49)	TNBC (*n* = 49)	HER2 (*n* = 26)	*P*-value
**Mean age at diagnosis** (years)	55	55	56	54	0.399
**Grade**					
Grade 1	12	0	1	0	<0.001*
Grade 2	75	24	15	4	
Grade 3	54	25	13	22	
**Tumour (T) stage**					
T1	31	8	13	5	0.107
T2	64	28	28	14	
T3	31	3	6	2	
T4	15	4	2	5	
**Nodal (N) status**					
N0	41	12	19	7	0.735
N1	43	14	11	9	
N2	39	16	10	8	
N3	18	7	9	2	
**NPI**					
1	35	16	8	23	0.619
2	65	27	14	17	
3	18	6	12	9	
**Stage**					
Stage 1	15	4	9	4	0.367
Stage 2	54	15	21	8	
Stage 3	72	30	19	14	
**Metastatic sites**					
Bone	103	38	26	9	0.353
Liver	85	28	29	13	
Pulmonary	50	16	24	13	
Brain	35	9	15	8	
Other	24	12	10	4	
TTR (Months)	56.0	48.4	26.9	34.3	0.380

*TNBC* triple negative breast cancer, *HER2* human epidermal growth factor receptor-2, *TTR* time to recurrence

^*^denotes statistical significance

### Survival analysis and patterns of metastatic breast cancer

OS was 24.2% (64/265) at mean follow up of 83.2 months (range 8.1—259.0 months). The mean time to recurrence (TTR) was 47.7 ± 38.5 months (range 3.0–194.3 months). The mean TTR for patients with LABC was 56.0 ± 41.3 months (range 3.0–190.0), 48.4 ± 41.1 months (range: 3.0–194.3 months) for LBBC, 26.9 ± 28.5 months (range: 3.0–132.6 months) for TNBC and 34.3 ± 21.8 months (range: 6.0–108.0 months) for patients with HER2 + disease (*P* < 0.001, one-way analysis of variance, †).

With regard to site of metastasis, within the LABC group, 73.0% had bone (103/141), and 60.3% had liver metastases (85/141). The bone was the most frequent initial site of recurrence (40.3%, 57/141), typically occurring at 64.3 months. Within the LBBC group, bone was also the most common site (77.6%, 38/49), which was followed by liver (57.1%, 28/49). For patients diagnosed with TNBC, the most common site was liver (59.2%, 29/49) and central nervous system (CNS) metastasis accounted for 30.6% (15/49). Similar patterns were observed for patients with the HER2 + molecular subtype with metastasis to liver, lung and CNS accounting for 46.2%, 50.0% and 42.3%, respectively (12/26, 13/26 & 11/26).

### Clinicopathological factors predicting breast cancer recurrence

Following univariable analysis, increased tumour grade (HR: 3.627, 95% CI: 1.940–6.77, *P* < 0.001), NPI (HR: 2.226, 95% CI: 1.385–3.576, *P* < 0.001), TNBC (HR: 1.927, 95% CI: 1.377–2.698, *P* < 0.001) and HER2 + molecular subtype (HR: 2.549, 95% CI: 1.661 – 3.912, *P* < 0.001) and receiving targeted therapy (HR: 1.541, 95% CI: 1.080–2.198, *P* = 0.006) all predicted shorter TTR. Invasive lobular carcinoma histological subtype (HR: 0.698, 95% CI: 0.503–0.969, *P* = 0.009), ER positivity (HR: 0.479, 95% CI: 0.366–0.620, *P* < 0.001), PgR positivity (HR: 0.659, 95% CI: 0.513–0.846, *P* = 0.010), *P* = 0.010) and receiving adjuvant endocrine therapy (AET) (HR: 0.540, 95% CI: 0.410–0.712, *P* = 0.001) predicted longer TTR. Table 2 outlines clinicopathological data not predicting TTR.

**Table 2:** Cox-regression analysis to determine predictive clinicopathological and treatment characteristics of patients likely to develop metastatic disease

Variable	Hazard ratio	95% Confidence interval	*P* value
**Age at diagnosis (years)**			
Age < 65	Reference		
Age > 65	1.141	0.858–1.517	0.365
**Menopausal status**			
Premenopausal	Reference		
Postmenopausal	1.0223	0.794–1.318	0.086
**Nottingham grade**			
Grade 1	Reference		
Grade 2	2.992	1.597–5.606	<0.001*
Grade 3	3.627	1.940–6.779	
**Histology**			
Ductal	Reference		
Lobular	0.698	0.503–0.969	0.009*
Other	0.994	0.682–1.447	
**Tumour stage**			
T1	Reference		
T2	1.219	0.888–1.674	0.380
T3	1.068	0.724–1.576	
T4	0.881	0.552–1.406	
**Stage**			
1	Reference		
2	1.294	0.848–1.974	0.400
3	1.316	0.877–1.974	
**NPI**			
1	Reference		
2	1.520	0.974–2.422	<0.001*
3	2.226	1.385–3.576	
**ER status**			
ER negativity	Reference		
ER positivity	0.479	0.366–0.620	<0.001*
**PgR status**			
PgR negativity	Reference		
PgR positivity	0.659	0.513–0.846	0.010*
**HER2 status**			
HER2 negativity	Reference		
HER2 positivity	1.170	0.824–1.548	0.270
**Molecular subtype**			
Luminal A	Reference		
Luminal B	1.019	0.719–1.444	<0.001*
TNBC	1.927	1.377–2.698	
HER2	2.549	1.661–3.912	
**Adjuvant chemotherapy**			
Did not receive	Reference		
Received	0.859	0.673–1.098	0.220
**Endocrine therapy**			
Did not receive	Reference		
Received	0.540	0.410–0.712	0.001*
**Targeted therapy**			
Did not receive	Reference		
Received trastuzumab	1.541	1.080–2.198	0.006*
**Radiotherapy**			
Did not receive	Reference		
Received	0.540	0.410–0.712	0.077

*NPI* Nottingham Prognostic Index, *ER* estrogen receptor, *PgR* progesterone receptor, *HER2* human epidermal growth factor receptor-2, *TNBC* triple negative breast cancer

^*^denotes statistical significance

**Fig. 1:**
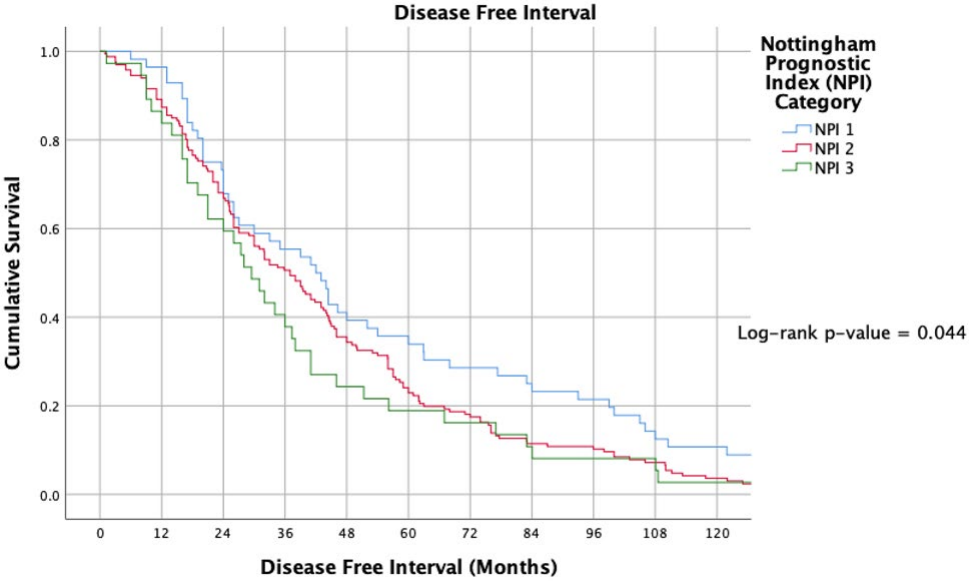
Timing of disease-free interval from timing of surgery for index breast cancer to metastasis according to Nottingham Prognostic Indices

Tumour grade correlated with TTR: Patients with grade 3 disease had a mean TTR of 40.0 ± 33.4 months, compared to 111.3 ± 60.3 months and 46.9 ± 33.6 months in grade 1 and grade 2 disease, respectively (*P* < 0.001, †). NPI correlated with TTR (Fig. 1): Patients with NPI category 3 (> 5.4) had a mean TTR of 43.3 ± 33.4 months, compared to 102.2 ± 23.9 months and 57.7 ± 49.8 months with NPI category 2 (3.4–5.4) and NPI category 1 (< 3.4) (*P* < 0.001, †).

**Fig. 2:**
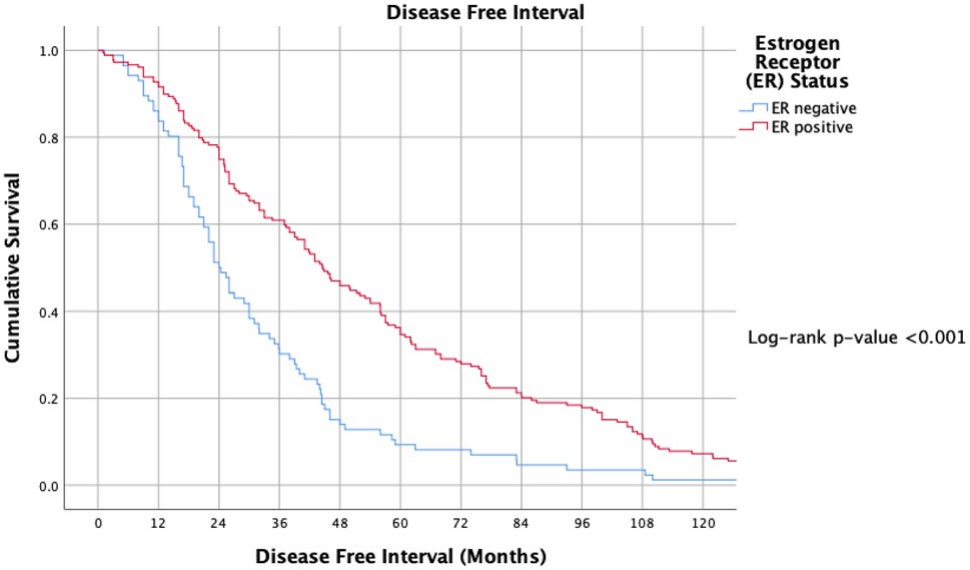
Timing of disease-free interval from timing of surgery for index breast cancer to metastasis according to oestrogen receptor status at the time of resection

**Fig. 3:**
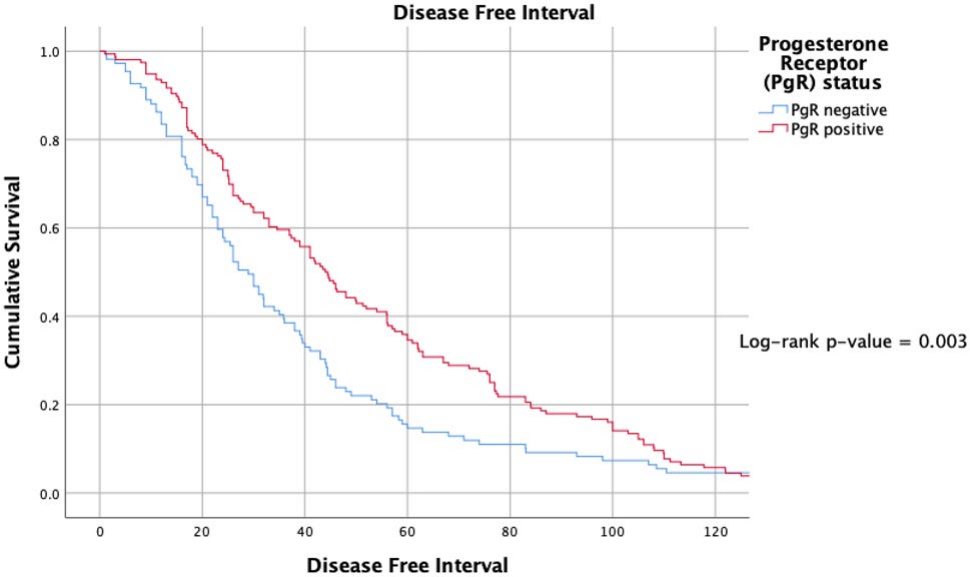
Timing of disease-free interval from timing of surgery for index breast cancer to metastasis according to progesterone receptor status at the time of resection

**Fig. 4:**
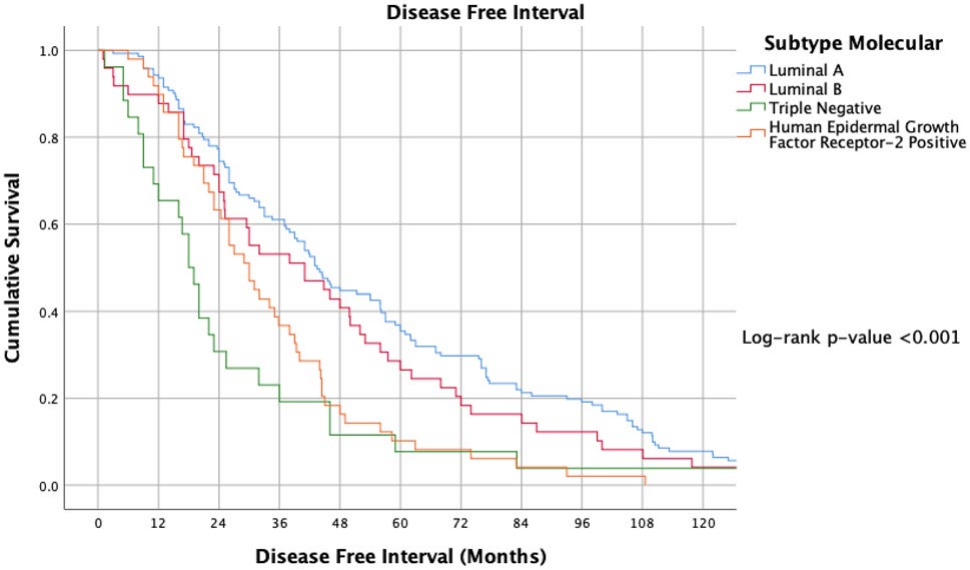
Timing of disease-free interval from timing of surgery for index breast cancer to metastasis according to molecular subtype

ER and PgR positivity were associated with longer TTR (log-rank *P* < 0.001 and *P* = 0.003) (Figs. 2 and 3). TTR was also impacted by molecular subtype; patients with luminal disease outperformed their TNBC and HER2 + counterparts in relation to DFI (*P* < 0.001) (Fig. 4).

### Adjuvant therapies predicting breast cancer recurrence

**Fig. 5:**
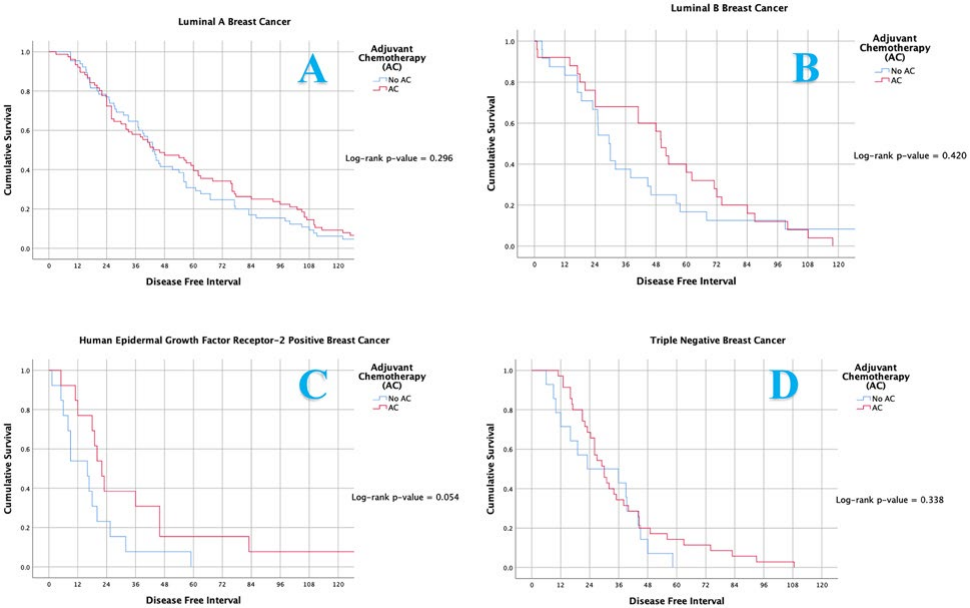
Timing of disease-free interval from timing of surgery for index breast cancer to metastasis according to molecular subtype for those treated with adjuvant chemotherapy and those spared treatment: (**A**) Luminal A breast cancer, (**B**) luminal B breast cancer, (**C**) human epidermal growth factor receptor-2 positive breast cancer and (**D**) triple negative breast cancer

Following index cancer diagnosis, 78.5% of patients underwent adjuvant radiotherapy (XRT) (208/265), 70.9% underwent AET (188/265) and 55.8% adjuvant chemotherapy (AC) (148/265). Overall, receipt of AET improved TTR (HR: 0.540, 95% CI: 0.410–0.712, *P* = 0.001). Neither adjuvant XRT or AC impacted TTR (Table 2). Those in receipt of Trastuzumab outperformed their counterparts (*P* = 0.006); furthermore those patients who were HER2 receptor positive who received Tratuzumab had improved TTR in comparison to those patients who were HER2 receptor positive who did not receive Tratuzumab (*P* = 0.023). The use of AC failed to influence TTR for all molecular subgroups (Fig. 5A–D); however AC prescription in TNBC trended towards longer TTR (*P* = 0.054) (Fig. 5C).

### Clinicopathological predictors of survival in breast cancer recurrence

**Fig. 6:**
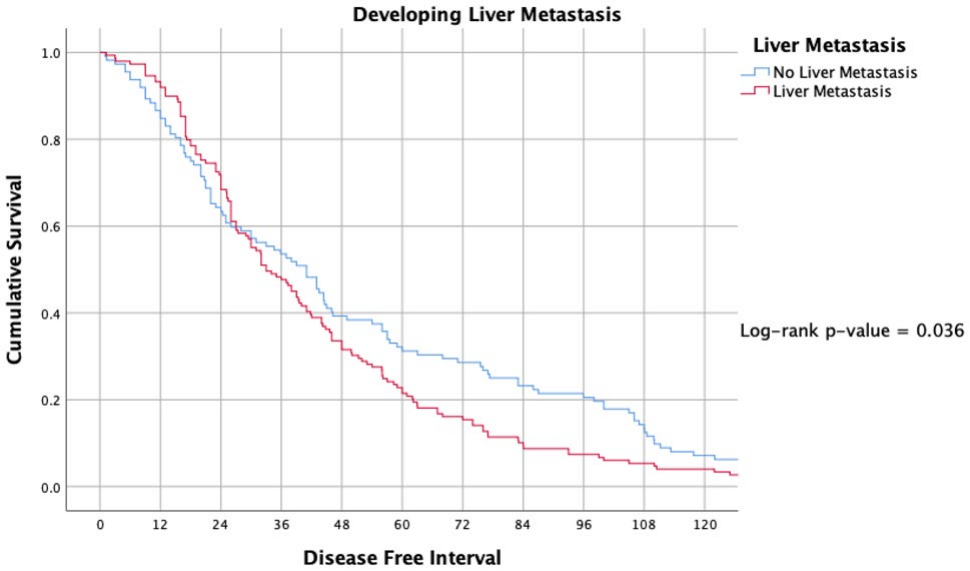
Kaplan Meier analysis assessing time to metastasis for those who developed hepatic metastasis versus those who did not

Being aged 65 years or older (HR: 1.444, 95% CI: 1.058–1.972, *P* = 0.021), developing liver metastasis (HR: 1.944, 95% CI: 1.432–2.639, *P* < 0.001) and receiving chemotherapy (HR: 1.446, 95% CI: 1.037–2.018, *P* = 0.027) predicted worse survival. Receiving endocrine therapy (HR: 0.668, 95% CI: 0.498 – 0.895, *P* = 0.007) and receiving Trastuzumab (HR: 0.559, 95% CI: 0.376–0.892, *P* = 0.003) predicted improved survival (Table 3). Developing bone, pulmonary or brain metastasis failed to independently predict worse clinical outcomes in this series (Table 3). Figure 6 illustrates the Kaplan Meier analysis for those who developed liver metastasis versus those who did not (*P* = 0.036).

**Table 3:** Clinicopathological predictors of Survival in Metastatic Breast Cancer

Variable	Hazard ratio	95% confidence interval	*P* value
**Age at diagnosis (years)**			
Age < 65	Reference	1.058–1.972	0.021*
Age > 65	1.444		
**Menopausal status**			
Premenopausal	Reference		
Postmenopausal	1.114	0.815–1.523	0.500
**Site of metastasis**			
Bone	0.798	0.590–1.079	0.140
Liver	1.944	1.432–2.639	<0.001*
Pulmonary	1.282	0.962–1.708	0.088
Brain	0.740	0.492–1.113	0.140
Other	1.030	0.714–1.486	0.870
**Treatment received**			
Chemotherapy	1.446	1.037–2.018	0.027*
Radiotherapy	0.098	0.726–1.374	0.999
Endocrine therapy	0.668	0.498–0.895	0.007*
Targeted therapy	0.559	0.376–0.852	0.003*

^*^denotes statistical significance

### Therapeutic strategies for metastatic disease

**Fig. 7:**
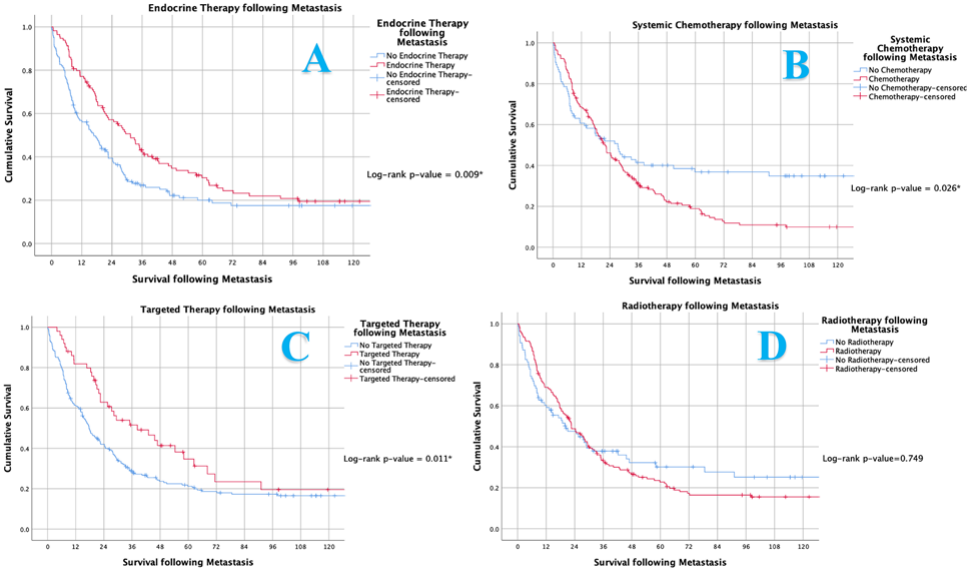
Kaplan Meier analysis assessing (**A**) endocrine therapy, (**B**) systemic chemotherapy, (**C**) targeted therapy and (**D**) radiotherapy and their impact on survival in those developing metastasis

**Fig. 8:**
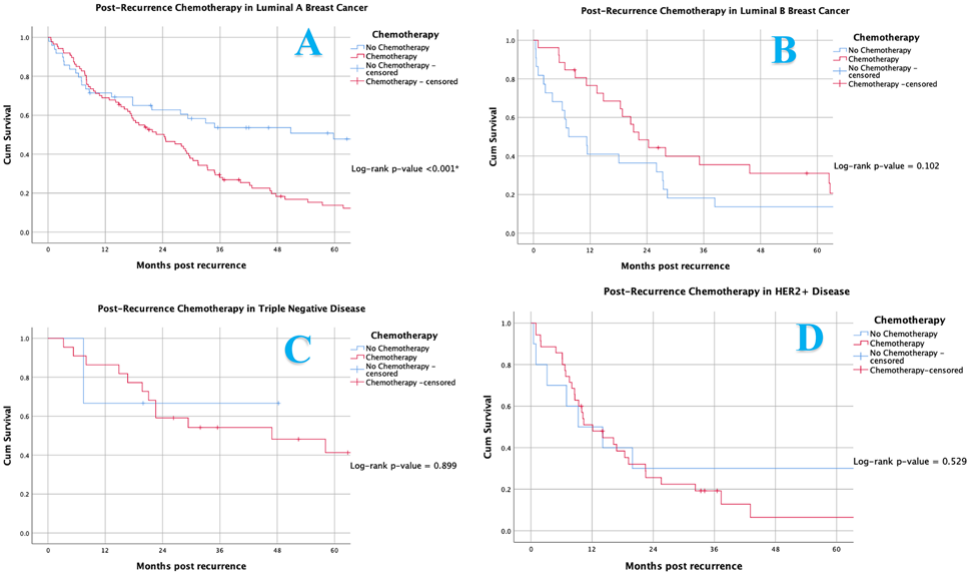
Kaplan Meier analysis assessing the impact of post-recurrence chemotherapy prescription in (**A**) luminal A breast cancer, (**B**) luminal B breast cancer, (**C**) human epidermal growth factor receptor-2 positive breast cancer and (**D**) triple negative breast cancer

Following metastatic development, 64.2% of patients received systemic chemotherapy (170/265), 62.3% received XRT (165/265), 43.0% received endocrine therapy (114/265) and 18.8% received targeted therapies (Trastuzumab) (50/265). In this setting, receiving endocrine therapy (*P* = 0.009), systemic chemotherapy (*P* = 0.026) and targeted therapy (*P* = 0.011) enhanced survival for patients; however radiotherapy had limited effect on survival (*P* = 0.749) (Fig. 7). Patients with recurrence of LABC derived no benefit from systemic chemotherapy prescription following metastasis (*P* < 0.001) (Fig. 8A). The impact of chemotherapy on other molecular subtypes are illustrated in Fig. 8B, C and D.

## Discussion

In this assessment of patients who suffered breast cancer recurrence, our primary aim was to determine the pattern of metastases and impact of molecular subtypes on outcome. Additionally, we hoped to address any host or tumour factors which may impact prognosis and outcome. The current analysis suggests a myriad of factors are predictive of metastatic dissemination, with molecular tumour features (such as intrinsic biological subtype and steroid hormone receptor status) and traditional parameters (such as Nottingham tumour grade and NPI) all predicting oncological outcome for these patients. Thus, the authors advocate for the introduction of formal screening and tailored treatment programmes for patients at high risk of recurrence, in attempt to control breast cancer related morbidity and mortality.

In this series, LABC constituted the largest cohort of patients, with the inherent tendency to initially metastasise to bone, with impending liver metastasis a recurring trend. This pattern is highlighted in several other large analyses, including one from our translational research facility [22, 23] describe a series of over 2000 patients with similar patterns of recurrence, while Ignatov et al. replicated these findings in their analysis of almost 15,000 patients with an index non-metastatic breast cancers [23–25]. Moreover, a similar pattern was observed in our series for those with LBBC. Conversely, those diagnosed with TNBC and HER2 + disease suffer shorter disease-free intervals and higher rates of visceral metastasis, with pulmonary, hepatic and CNS metastasis dominating these patient cohorts. Both series from Katz and Ignatov further validate these trends in disease recurrence, echoing the results of the current analysis; TNBC and HER2 + molecular subtypes harbour more aggressive neoplasia than patient with luminal disease, with greater affinity for visceral metastasis and poorer oncological outcome [23–25]

Data from the current analysis implicates metastatic LABC favour bone metastases, carrying better prognoses when compared to other breast cancer molecular subtypes [26–28], with recurrence intervals occurring as late as decade after treatment of their index cancer. Furthermore, patients with ‘true’ LABC demonstrate excellent responses to endocrine therapies, as illustrated in this analysis [29]. In contrast, steroid hormone receptor negative cancers relapse within 3–5 years of their initial therapy [13, 30]. Thus, oncological screening seems more effective at targeting those with TNBC or HER2 + molecular subtypes, particularly if surveillance strategies may be tailored to be site specific. Recent analyses suggest TNBC cancers have an almost sevenfold propensity of propagating lung metastasis compared to their LABC counterparts [31–33]. Moreover, Perou et al. highlight the recurring trend of hepatic metastasis within the context of HER2 + and TNBC disease, with combined metastases to both liver and bone occurring more frequently than skeletal metastases in isolation [34]. Smid et al. highlight the penetrance of brain metastasis in HER2 + and TNBC disease [35], which is supported by data from Hicks et al. which implicates the propagation of CNS metastases to occur in the setting of ER negative, basal cytokeratin 5/6 expressing cancers, or in those overexpressing HER2/ErbB receptor tyrosine kinase [36]. This is perhaps somewhat unsurprising within the context of HER2 + disease, due to the propensity of lipophilic HER + cells to disseminate across the lipid neutral blood brain barrier [37], in tandem with the inability of Trastuzumab to cross this biological barrier [38–40]. Within the TNBC and HER2 + paradigms, the authors also wish to acknowledge the worrying observation in our analysis of vital organs being targeted for metastasis at short disease-free intervals. This pattern replicates the work of Ribelles et al. who illustrated the disease free interval of HER2 + metastatic disease was shorter than other molecular subtypes [41], as well as Liedtke et al., who described the initial 3 years post-TNBC diagnoses as the critical, high-risk period for recurrence [42]. Thus, the authors believe the emergence and incorporation of targeted screening strategies into the metastatic breast cancer programme would prove profusely advantageous in attempt to control distant metastases.

However, the crux of implementing tailored screening programmes rely on the identification of predictors of visceral recurrence in TNBC, HER2 + and high-risk luminal epithelial cancers. Ren et al. suggested traditional clinicopathological biomarkers have the potential to inform prognosis [9]; Nottingham tumour grade, histological subtype, NPI, intrinsic molecular subtyping and steroid hormone receptor status all informed duration of remission in our series. This is unsurprising as the aforementioned tumour characteristics are renowned, crucial factors responsible for driving tumourgenesis and concomitant metastasis [9, 43]. Overall, there is a vogue towards emphasising molecular tumour properties to inform prognoses and mortality [33, 44, 45]; however the results of the current analysis support both histopathological and immunohistochemical predictors of informing metastases. As we enter the era of precision oncology, it is imperative that we recognise the inherent value of traditional and novel taxonomic markers of disease within breast cancer.

In this series, diagnoses of TNBC and developing hepatic metastasis negatively impacted overall survival. Data from this study highlights that once a patient developed hepatic metastasis, only 60% of patients remained alive at 24 months. Liver metastasis from breast cancer occurs due to haematological dissemination [46], allowing the disease to be labelled as ‘systemic’ in such incidences. This explains why less than 5% of such metastasis present with hepatic involvement in isolation [47]. Moreover, within TNBC, liver metastasis is renowned to carry dismal outcomes [48, 49], often leading to palliation and the prioritisation of symptomatic management and control. Palliative locoregional measures to control liver metastasis are mooted and include transarterial embolization, transarterial chemoembolization, interstitial brachytherapy and selective internal radiotherapy [50]. In recent times, there has been a vogue towards addressing the possibility of curing oligometastatic breast cancer [51], although gains have been minimal, with robust systemic chemotherapy prescription still dominating therapy, as demonstrated in the results of this analysis. Furthermore, results from the current analysis promote selective de-escalation of aggressive therapeutic strategies within the setting of metastatic LABC, as evident in Fig. 8A, and advocate for the further personalising of oncological treatment from current robust measures.

Overall, while enhancing patient survival remains the priority, the introduction of a tailored surveillance programme based on prognostic indicators is imperative. Tailored screening using prognostic factors, such as molecular subtype, tumour grade and NPI could be critical in identifying at risk patients while incorporating circulating biomarkers into the paradigm carries promise [52]. Data from the current study supports the previous recommended guidelines by the American Society of Clinical Oncology (ASCO), National Comprehensive Cancer Network (NCCN) and European Society for Medical Oncology (ESMO) to regularly screen for disease recurrence [53]; although while measurement of oncological outcome is a simple metric used in cancer survivors, we must also acknowledge that improving quality of life and survivorship outcomes is paramount, something not addressed in the current analysis.

## Conclusion

This analysis suggests that readily available clinicopathological and treatment parameters may predict patients at an increased risk of disease recurrence through metastatic dissemination. The prospect of developing a tailored screening program to identify those at the greatest risk of recurrence is crucial as we attempt to control breast cancer from becoming a systemic disease with catastrophic outcomes surrounding patient morbidity and mortality.
